# Modelling the emergence of rodent filial huddling from physiological huddling

**DOI:** 10.1098/rsos.170885

**Published:** 2017-11-22

**Authors:** Stuart P. Wilson

**Affiliations:** 1 Department of Psychology, The University of Sheffield, Sheffield, UK; 2 Sheffield Robotics, The University of Sheffield, Sheffield, UK

**Keywords:** thermoregulation, self-organization, metabolism, entropy, huddling, brown adipose fat tissue

## Abstract

Huddling behaviour in neonatal rodents reduces the metabolic costs of physiological thermoregulation. However, animals continue to huddle into adulthood, at ambient temperatures where they are able to sustain a basal metabolism in isolation from the huddle. This ‘filial huddling’ in older animals is known to be guided by olfactory rather than thermal cues. The present study aimed to test whether thermally rewarding contacts between young mice, experienced when thermogenesis in brown adipose fat tissue (BAT) is highest, could give rise to olfactory preferences that persist as filial huddling interactions in adults. To this end, a simple model was constructed to fit existing data on the development of mouse thermal physiology and behaviour. The form of the model that emerged yields a remarkable explanation for filial huddling; associative learning maintains huddling into adulthood via processes that reduce thermodynamic entropy from BAT metabolism and increase information about social ordering among littermates.

## 1. Introduction

Social thermoregulation has been described in social insects, reptiles, birds and mammals, including humans. For example, honeybee swarms cluster to form structures whose surface density varies with the environment temperature, maintaining the porous core at thermal homeostasis [1]. Common garter snakes in Canada have been found to hibernate in dens comprising 8000 animals for up to a third of the year during winter [2]. Emperor penguins aggregate at a density of up to 10 birds per square metre, adapting the overall shape of the huddle to weather cold winds [3]. Bats huddle to compensate for poor insulation in forest roosting sites, and huddling is important for the initiation and maintenance of group cohesion during collective roost-switching behaviours [4]. Other rodents, including mice [5], rats [6], rabbits [7] and degus [8], huddle to insulate from the cold, by collectively reducing the exposed surface-area-to-volume ratio of the group [9,10]. And social thermoregulation in primates (bamboo lemurs) has recently been shown to be more important for temperature homeostasis than the choice of resting site [11].

The huddling behaviour of laboratory rodents, in particular rats and mice, has emerged as a model system for the study of social thermoregulation. This is due to their prevalence as laboratory species, and to the precision with which the thermal physiology of these species has been investigated [12,13]. A number of agent-based computer models [5,8,14–20] have been formulated to describe how rodent huddling behaviours at the level of the group emerge from simple rules of interaction between individuals [5,6,21,22].

The individual behaviour from which rodent huddling emerges has been described formally as ‘homeothermotaxis’; turning in the direction that brings the body temperature closer to a preferred temperature [18,19]. As such, individuals act like the magnetic spins in an Ising model, or the particles in a Vicsek model from statistical physics, attracted or repelled by the relative body temperatures of their littermates [20]. These thermodynamic models have been used to explain a phase transition in mouse huddling behaviours, from aggregation in cold environments to dispersion at warmer temperatures [5,18], and by analogy with particle systems they generate new predictions, such as a temperature-dependent peak in ‘pup flow’, where animals cycle between the cold huddle periphery and its warm core [6,20].

Central to thermodynamic descriptions of huddling is a role for brown adipose fat tissue (BAT), which evolved as the organ of thermogenesis in early mammals [23,24], and has been shown to be a requirement for the emergence of huddling [25–28]. Individual differences in early huddling can be explained in terms of individual differences in thermal physiology, for example sex differences in rat huddling can be understood by considering females, who are born with more BAT, as heat sources and males as heat sinks [29]. Syrian golden hamsters, which start to produce heat by BAT thermogenesis relatively late in postnatal development, will initiate huddling behaviours when introduced into groups of age-matched rats, who are generating heat via BAT at this age [27,26]. In turn, early individual differences in huddling behaviours have been shown to predict metrics of adult social behaviour. For example, rabbit pups that tend to occupy peripheral versus central positions in the huddle during the first postnatal week are more likely to jump a gap to attend to the cries of a distressed littermate when tested as adults ([30]; see also [31–33]). In similar terms, social thermoregulation is thought to confer, in a number of species, human-like social behaviours [34,35], such as the ‘contact comfort’ offered in consolation to distressed conspecifics by prairie voles [36], bonobos [37] and chimpanzees [38].

As precocial mammals develop, maximal energy in BAT stores at birth decay, as does the drive towards huddling based on physiological demands [6,24,26,39,40]. However, rodents continue to huddle into adulthood, and the transition from physiological huddling to ‘filial huddling’ is characterized by a preference for (even cool) objects whose odours have been associated with a warm soft touch [6,39,41–44]. According to Alberts [45]: ‘The olfactory-perceptual preferences that direct and maintain social contact (huddling) behavior in rat pups are established by the association of olfactory cues with the thermotactile stimulation present during mother-litter interactions. The induction of odor preferences that guide affiliative social behaviour are not susceptible to other reinforcers such as suckling rewards.’ Thus, associative learning, and in particular odour-heat conditioning, is thought to play a primary role in the emergence of rodent social behaviour.

Emerging theories of social thermoregulation in primates are beginning to extrapolate from the data on rodent huddling to explain how complex human psychological concepts, such as the establishment of distinct attachment styles, and the formation of internal models for social behaviour, may be supported by the same neural systems that regulate huddling behaviours in rats and mice [34], such as hypothalamic circuitry and oxytocin regulation [6,35,46]. A central theme in these discussions is that neural systems which evolved under selection pressure to allow animals to *react* to thermal stimuli, to help minimize the energetic costs of physiological thermoregulation, have since been supplanted by more elaborate (presumably neocortical) circuitry that enables animals to *predict* the thermal consequences of contact with conspecifics [34]. The idea has recently been developed further by Morrison [35], who proposes that ‘[Neural] pathways involved in social thermoregulation may have evolved to use conspecific touch patterns as shorthand for “warm and close”. […Such] temporal and contextual shorthand may manifest in regulatory neural shortcuts in which “warm and close” states can be instigated by social touch alone, rather than requiring a cycle of behaviorally mediated physical warmth restoration following actual temperature decreases.’ Essentially, the neural substrate of social thermoregulation may provide a scaffold for the emergence of non-thermal social behaviours. In rodents, this may occur during postnatal development, as odour-heat conditioning between huddling littermates replaces the physiological drive for huddling with a drive based on the strength of association to olfactory cues.

To make explicit these assumptions about the role of odour-heat conditioning in social thermoregulation, and to test the idea that social thermoregulation can provide a scaffold for the development of non-thermal social behaviours, the current study presents a model, based on the thermodynamic description of rodent huddling by Wilson [20], which predicts how huddling behaviours should change over time as BAT physiology matures in litters of mice. The model is then extended to ask how odour-heat conditioning during contact in the huddle shapes the emergent group behaviour. The model is calibrated to data on the development of thermal physiology and individual thermal behaviour in mice, and thus it may be used to generate testable predictions about the developmental time course of the emergence of social behaviour.

## 2. Models

A Monte Carlo algorithm has recently been shown to capture the statistics of huddling behaviour [20], i.e. the distribution of groups of pups in contact, as predicted by more elaborate models of the underlying physical interactions between littermates (see [18,19,47]). The idea is to iteratively reconfigure the distribution of pups between groups by choosing pairs of pups at random from the litter and either joining together the groups to which they belong, or isolating one from its group. The decision between these two alternatives is made by comparing a randomly generated number each time to a value representing the probability that those two pups will remain in contact. If the random number is less than the probability of remaining in contact, then the groups to which the two pups belong are joined together to form a larger group; else one pup is isolated to form a new group of size 1. In this way, a higher probability makes larger huddles more likely to form.

More precisely, the huddling algorithm involves iteratively selecting a pup at random, *a*, then selecting a second pup at random from a different group, *b*, and then either joining together the groups to which pups *a* and *b* belong with probability *ρ*_*a*,*b*_, or detaching *a* from its group to form a new group of size 1, where

2.1 $$\rho_{a,b}={\left(1+e^{-T}\right)}^{-1},$$

and the threshold *T* is referred to as the ‘temperature parameter’. Predicting how huddling statistics change over developmental time thus requires a definition of how *T*, which determines the likelihood of two groups joining to form a larger huddle, changes as the thermal physiology matures.

Although studies of the developing thermal physiology of mice are numerous, few have quantified the relationship between the maturing thermal physiology *and* the development of thermal behaviour in the same animals and under the same experimental conditions. The basis of the model is therefore a dataset published by Eedy & Ogilvie [48], which describes, for the same set of mice, how the body mass, the mass-specific metabolic rate and crucially the preferred ambient temperature of the animals change across the first 60 postnatal days. In that study, animals were briefly isolated from the huddle on successive days and placed on a thermocline, i.e. an apparatus in which temperature varies continuously from hot on one side to cold on the other. The recorded temperature of the location at which the animals settled is referred to herein as the preferred ambient temperature.

The next section shows how, together with existing data describing changes in mouse BAT over a similar period [49], the preferred ambient temperature of developing mice can be related to the changing thermal physiology, and thus how the physiological drive towards huddling, *T*, develops. To investigate the transition from physiological to filial huddling, the data of [49,48] were first approximated by a series of simple expressions to estimate the preferred ambient temperature *T*; then values of *T* were fed through the huddling model (equation (2.1)) to predict how group sizes change during postnatal development.

Corresponding to a ‘no-learning’ control condition, the algorithm begins with *n*=7 pups joined in a single group, and then evaluates equation (2.1) many thousands of times, comparing *ρ*_*a*,*b*_ to a random number between 0 and 1 each time to determine the reconfiguration of the pups between subgroups, while *T* changes according to the fit to the preferred ambient temperature measured by Eedy & Ogilvie [48]. Corresponding to the main ‘learning’ condition, the same procedure is repeated, using an associative learning rule to adapt a set of associative strengths between pups, and in turn to weight *T* by these associative strengths, on each iteration. This extension is detailed in the final Results section and represents the ability of animals to learn, by odour-heat conditioning, to predict the outcome of future contacts.

A full implementation of the model in C++ is included as electronic supplementary material, S1, together with a script, written in Python, to extract and display the changing huddling statistics, i.e. the average group size predicted on each postnatal day.

## 3. Results

### 3.1. Metabolism as the entropy of brown adipose fat tissue energy consumption

**Figure 1.**
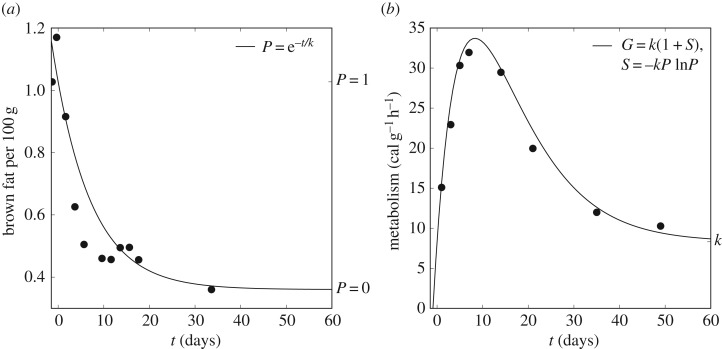
Development of mouse physiology. Data recorded by Lagerspetz [49] in (*a*) and Eedy & Ogilvie [48] in (*b*) are shown as black dots. Curves show the fit of the model. (*a*) The depletion of brown adipose fat reserves was modelled as an exponential decay, *P*, with a time constant of *k*=8.31. (*b*) The mass-specific metabolic rate, *G*, was modelled in terms of the entropy, *S*, associated with *P*. According to this model, the mass-specific metabolic rate is predicted to peak at postnatal day *k*=8.31.

The points *p* plotted in figure 1*a* show the weights of BAT, as a percentage of the body weight, as measured in mice at different ages by Lagerspetz [49]. Using the first (*p*_1_) and final (*p*_0_) measurements to define *p*^′^=(*p*−*p*_0_)/(*p*_1_−*p*_0_), the rescaled points *p*^′^ were well approximated by an exponential decay,

3.1 $$P=e^{-t/k},$$

with *k* a time constant describing the rate of depletion of BAT reserves.

Figure 1*b* shows the mass-specific metabolic rate (converted to calories per gram per hour) reported by Eedy & Ogilvie [48], and a fit to these data by *G*, defined as

3.2 S=−kP ln⁡P

and

3.3 G=k(1+S),

where the unit term corresponds to the basal metabolic rate and *S* is the entropy associated with *P*. As such, *k* is a unit of energy, and the fit obtained using *k*=8.31 ascribes particular significance to *k* as the universal gas constant [50]. Thus the mass-specific metabolic rate is proposed to be the thermodynamic entropy generated by the conversion of energy that is stored in BAT at birth.

Entropy is a measure of disorder, describing the number of different configurations of a system that are possible. Only in open systems can entropy decrease, i.e. in systems that exchange matter with the surrounding environment. From day 8, figure 1 shows a decrease in the entropy (per unit mass) that relates the amount of BAT to the rate of metabolism. This suggests that as the mouse uses energy stored in BAT, and exchanges matter with its environment via respiration, the number of possible states of the brown fat tissue decreases from day 8. Intuitively, the effect is similar to the way that water molecules become more ordered when they freeze to create ice.

### 3.2. Behavioural thermoregulation as a competition between fat and muscle

**Figure 2.**
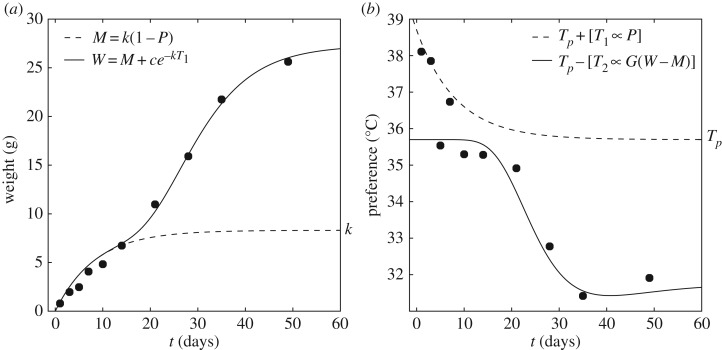
Development of mouse behavioural thermoregulation. Data recorded by Eedy & Ogilvie [48] are shown as black dots. Curves show the fit of the model. (*a*) The growth curve was modelled as a muscle mass *M*, plus a non-muscle mass that rises as temperature *T*_1_ falls. (*b*) The environment temperature selected by mice on a thermocline was modelled as a combination of temperatures *T*_1_ and *T*_2_, related to the development of muscle and non-muscle masses, respectively. The model suggests that behavioural thermoregulation (*b*) is driven by the developing metabolism, defined in terms of the entropy associated with brown adipose fat depletion.

The growth curves of mice typically display an initial rise (of decreasing magnitude) followed by the more familiar sigmoidal shape that is often modelled as a logistic/autocatalytic function (e.g. [51–53]; see electronic supplementary material, figure S2). Figure 2*a* shows that the growth curves measured by Eedy & Ogilvie [48] are well described as sums of two quantities,

3.4 M=k(1−P)

and

3.5 $$W=M+c\;e^{-kT_{1}},$$

where *c*=19 is a constant (see electronic supplementary material, figure S2) and *T*_1_ is a temperature.

Viewed in terms of the growth *rates*, the growth curve comprises a mass *M* that is driven towards a saturating value of *k* by *dM*/*dt*=*k*−*M*, and a second mass *N*=*W*−*M*, which increases with *M* at a rate that decreases as *M*→*k*, according to *dN*/*dM*∝*N*. The initial rise in *N* occurs at around postnatal day 15, which for mice coincides with a rapid development of muscle shivering as a response to cold challenge [49]. The quantity *M* is thus interpreted to be the muscle mass and the quantity *N* is therefore referred to as the non-muscle mass.

The temperatures selected by the developing mice (when isolated) on a thermocline, as recorded by Eedy & Ogilvie [48], are shown in figure 2*b*. Selected temperatures could also be approximated by a combination of two curves, when added to a ‘target’ adult body temperature assumed to be *T*_*p*_≈36^°^*C*:

3.6 $$T_{1}\propto P$$

and

3.7 $$T_{2}\propto GN,$$

where the constants of proportionality were estimated as *c*_1_=3 and *c*_2_=0.025, respectively.

On each postnatal day, the animals appear to select either *T*_*p*_+*T*_1_ or *T*_*p*_−*T*_2_, suggesting that thermoregulatory systems based on BAT energy metabolism (responsible for *T*_1_) and total (non-muscle) metabolism (responsible for *T*_2_) are in competition for behavioural thermoregulation. Compared with the dominance of BAT in determining the preferred temperature in the first few postnatal days, the later dominance of other thermoregulatory systems represented by *T*_2_ may correspond with increases in e.g. liver, thyroid and adrenal activity [49].

### 3.3. From physiological huddling to filial huddling

**Figure 3.**
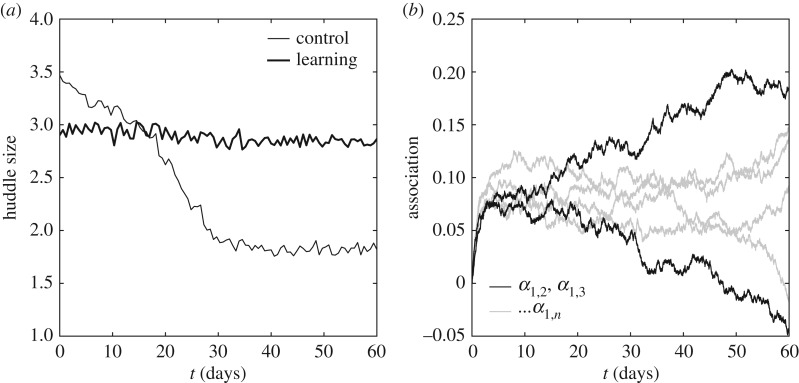
From physiological huddling to filial huddling. (*a*) Huddling statistics were generated by supplying a Monte Carlo model [20] with temperatures *T*=*T*_1_− *T*_2_, weighted either by *α*=1 to define a ‘physiological huddling’ control condition, or by allowing associative strengths *α*_*a*,*b*_ to adapt. In the control condition, the predicted mean group size decreases with *T* (light trace), but with associative learning enabled (heavy trace) high huddling levels remain stable into adulthood, with huddling dominated by ‘filial’ preferences (based on *α*) rather than physiology (based on *T*) from approximately day 15. (*b*) Development of the associative strengths of an arbitrary pup to the odours of its littermates in the ‘learning’ condition. Heavier traces highlight the emergent heterogeneity of filial huddling preferences (litter size; *n*=7).

The Monte Carlo method developed by Wilson [20] (equation (2.1)) can be used to translate *T*_1_ and *T*_2_ into a prediction of how the mean group size varies with age as the physiology matures from ectothermy to endothermy, using *T*∝*T*_1_−*T*_2_. Figure 3*a* (light trace) confirms that huddling (average group size) is predicted to decrease as the preferred temperature falls.

To model how pups learn from huddling interactions, each is assumed to maintain a strength of association *α* for the odour of each of its littermates. The thermodynamic temperature parameter *T* is then weighted at each time step by the strength of association of pup *b* for the odour of pup *a*,

3.8 $$T=\alpha_{b,a}(T_{1}-T_{2})\beta ,$$

where *β*=0.2 is an arbitrary scaling constant. Note that in the control condition, where huddling interactions are determined only by the maturing physiology, *α*=1 for all strengths of association.

The Delta rule from animal learning theory [54] can then be used to modify these associative strengths, treating each simulated encounter between *a* and *b* as a conditioning trial. On each iteration of the huddling algorithm, the strength of association of pup *a* for the odour of pup *b*, *α*_*a*,*b*_, is updated to better predict whether future contacts between them will be maintained,

3.9 $$\Delta \alpha_{a,b}=\gamma \left(r-\sum_{i\neq a}\alpha_{a,i}\right),$$

where the reward is *r*≡1 whenever the groups of *a* and *b* are combined, or *r*≡0 otherwise, and the learning rate is set to *γ*=0.001.

Figure 3*a* (heavy trace) shows that when littermates can learn to predict the outcome of huddling interactions, large huddles persist into adulthood, with larger huddles predicted from around day 15 in littermates able to learn compared to non-learning controls. Figure 3*b* shows the associative strengths learnt by an arbitrary pup, which grow to an early peak for all littermates, but thereafter increasingly discriminate among the littermates. The development of two associative strengths are highlighted, showing how littermates can learn strong positive or negative associations to one another. This emergent heterogeneity of preferences for maintaining contacts with specific littermates may constitute the basis of a rudimentary social system.

## 4. Discussion

The quantities and expressions derived in modelling the data of [48,49] suggest the following about the development of the thermal physiology and behaviour of the mouse. Mice are born with maximum BAT stores, which decrease exponentially over time as *P*=e^−*t*/*k*^, as is the typical profile for a precocial mammal [23]. From birth, mice accumulate body mass by a process that converts between fat and muscle, such that muscle mass increases as *M*=*k*(1−*P*). As such, the unit of energy *k* may be interpreted as a conversion factor between the (mass-specific) energy stored in fat tissue and the (absolute) muscle mass. The energy released in the conversion between fat and muscle is reflected by a spike in the entropy of BAT, measured as a peak in the mass-specific metabolic rate, i.e. the oxygen consumption. This spike is similar to the fluctuation in entropy (or heat capacity) measured by physicists during a Schottky anomaly (see [50,55]), or as occurs when an open system undergoes a transition from a low-energy ground state to a steady state of higher energy [56]. Here, the ground state corresponds to ectothermy, and the transition is to the higher-energy thermal physiology associated with endothermy. Ectothermy in the newborn makes it highly resistant to a cold environment, for example neonatal mice can recover from hypothermia at 0^°^*C*, whereas adults cannot [49]. However, the later transition to endothermy, through a peak in entropy, is required in order for the adult to sustain a higher energy metabolism; In the words of Erwin Schrödinger ‘…the higher temperature of the warm-blooded animal includes the advantage of enabling it to get rid of its entropy at a quicker rate, so that it can afford a more intense life process’ [57].

The results of adding odour-heat conditioning to the model of [20] provides a parsimonious explanation for the persistence of rodent filial huddling into adulthood, confirming the intuitions of several researchers (e.g. [34,35,45]). Equation (3.9) makes explicit the distinction between *reactive* behaviour (i.e. physiological huddling with all *α*=1; figure 3*a*, ‘control’) and *predictive* behaviour (i.e. filial huddling based on a learnt distribution of *α* values among littermates; figure 3*a*, ‘learning’). More detailed models of associative learning are expected to yield qualitatively similar results.

Beyond the *a priori* prediction that odour-heat conditioning might lead to the persistence of huddling into adulthood, the additional prediction that associative strengths among the litter can develop to be increasingly positive *or* negative was not anticipated, although the effect is simple to understand *post hoc*. Once two pups have developed a moderate association, an early contact that by chance results in a surprising outcome will lead to an adjustment in association that makes that predicted outcome less likely to result from future encounters. For example, two pups that expect to maintain contact (*α*>0) but do not, will adjust *α* to predict that future contacts are unlikely to be maintained (Δ*α*<0), and in doing so reduce the probability of maintaining future contacts. Steady increases or steady decreases in associative strength between pups (figure 3*b*) thus reflect an amplification of early individual differences between them and result from closing the loop between behaviour (equation (2.1)) and learning (equation (3.9)). By driving the summation term in equation (3.9) towards binary values of *r*, the overall associative strengths among the group remain bounded, thus enforcing a competition where pairwise increases and decreases in associative strength are balanced. The net effect is a steady state in which the system remains poised in a critical regime (*ρ*≈0.5).

These dynamics demonstrate the usefulness of computational modelling as a tool for teasing out the predictions of how even simple systems behave when learning is allowed to interact with behaviour. The new prediction in this case is that while associative learning enables huddling to persist into adulthood, individuals will become increasingly selective in their choice of huddling partners. This prediction could in principle be tested by measuring the changing preferences of animals, at thermoneutral temperatures, to huddle with surrogate objects scented with different littermate odours in a forced-choice experimental set-up. In similar set-ups using artificial odours, rats have been shown to spend more time on postnatal day 6 exploring odours recently paired with the experience of warmth, whereas on days 7 and 8 rats spend less time exploring an odour paired with the experience of cold, compared to baseline times spent exploring the warmer of two odour-neutral locations [44].

Filial huddling preferences can be induced by exposure to novel odours when paired with soft contacts and/or warm environments (rather than food [58]). The efficacy with which the association between odour and a warm soft touch can induce filial responses increases from birth [59] during the first two postnatal weeks [41], affecting huddling behaviours until around day 15 [45]. Consistent with these data, the point at which the two traces in figure 3*a* intersect defines a transition from physiological to filial huddling, i.e. at around postnatal day 15.

Day 15 represents a landmark in the development of rat social thermoregulation. Preferences measured in rats on day 15 can be induced by a single 2 h exposure at postnatal day 14 to a scented object other than the mother, only if it is both warm and soft (consistent with the definition of *r*), suggesting that a combination of the thermal and tactile properties of a contact determine its valence as a reward for odour conditioning. The emergence of filial huddling shown in figure 3*a* is thus consistent with the general finding that preferences for conspecifics are stronger for older (15–20 days) animals than younger animals (5–10 days), and are disrupted in older animals to a greater extent by blocking olfaction (e.g. by intranasal infusion of zinc sulphate) [39]. An important test of the model will be to establish whether day 15 is also a landmark in the development of mouse social behaviour.

According to the model, mechanisms of classical conditioning, which presumably involve the pre-optic region of the hypothalamus [23,24,46,60–62] and the olfactory bulb [39], adjust the relative strengths of association to the smells of littermates to better predict the thermal consequences of huddling encounters. As a result, huddling levels are maintained into adulthood, despite a decline in the immediate energetic benefits to huddling. In the process, the thermodynamic entropy is essentially converted into an information-theoretic entropy, storing among the relative strengths of association information about the history of rewarding and non-rewarding thermotactile experiences between littermates. This information represents a potential template for an adult social system in which animals prefer to interact with certain others.

## Supplementary Material

Modelling the emergence of filial huddling

## Supplementary Material

Analysis of huddling statistics

## Supplementary Material

Fitted growth curves for mice conceived and reared at different environment temperatures by Yamauchi (1983)

## Supplementary Material

Monte Carlo huddling modelling

## Data accessibility

The datasets supporting this article have been uploaded as part of the electronic supplementary material.
