# Supplementary figures and images for: Modelling the emergence of rodent filial huddling from physiological huddling
Source: R Soc Open Sci. 2017 Nov 22;4(11):170885. doi: 10.1098/rsos.170885 (PMC5717655; doi:10.1098/rsos.170885)

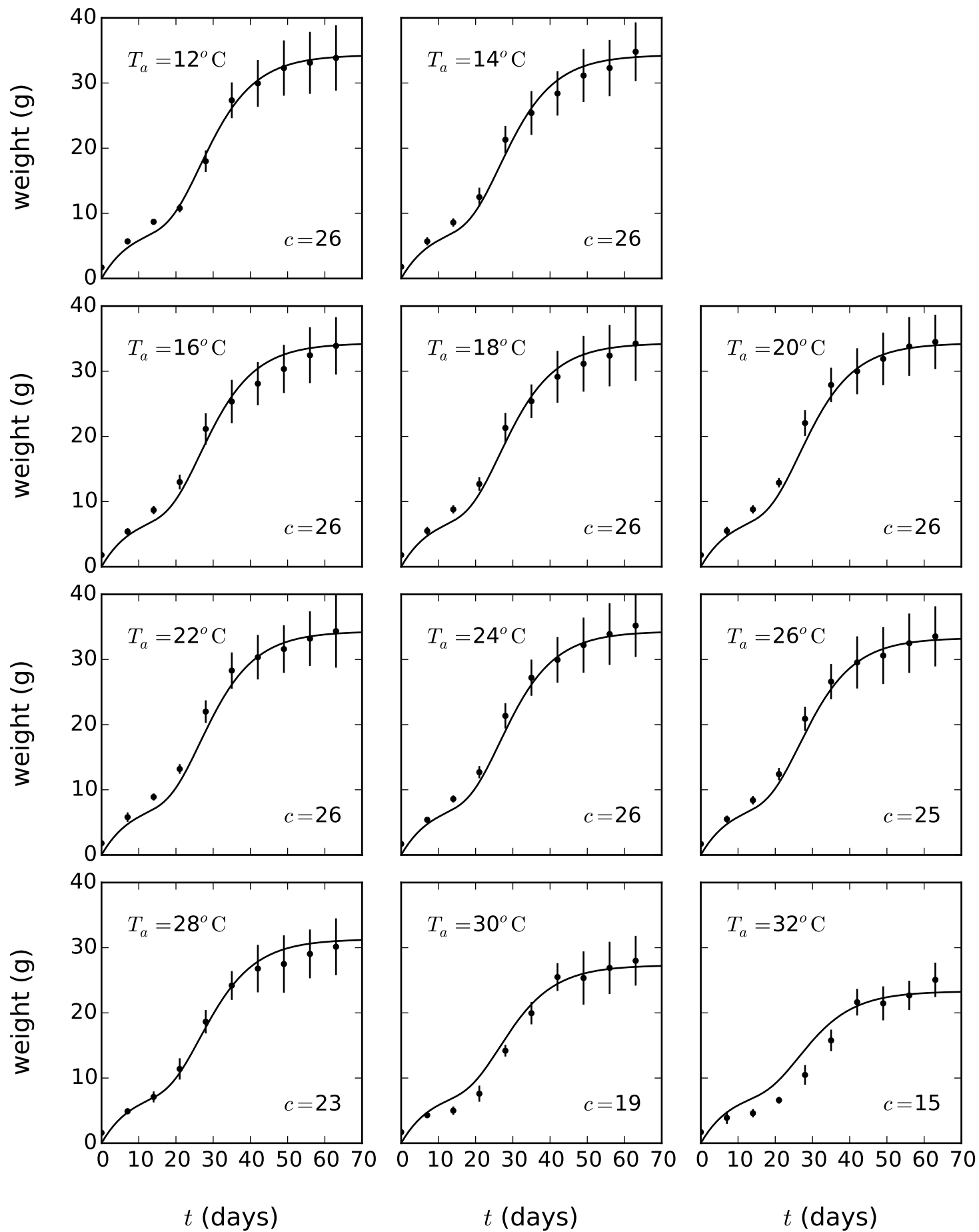

Supplement: Fitted growth curves for mice conceived and reared at different environment temperatures by Yamauchi (1983) [file rsos170885supp3.pdf]
